# Thoracocentèse versus drainage thoracique percutané dans le traitement des empyèmes thoraciques non tuberculeux de grande abondance: étude prospective et comparative préliminaire

**Published:** 2012-09-17

**Authors:** Eric Walter Pefura Yone, Christopher Kuaban, Léonie Simo

**Affiliations:** 1 Département de médecine interne et spécialités-Faculté de Médecine et des Sciences Biomédicales-Université de Yaoundé I/ Service de Pneumologie-Hôpital Jamot de Yaoundé; 2 Direction de la lutte contre la maladie, Ministère de la Santé Publique du Cameroun

**Keywords:** Thoracocentèse, drainage thoracique, empyème thoracique, adultes, Cameroun, Thoracentesis, thoracic drainage, thoracic empyema, adults, Cameroon

## Abstract

**Introduction:**

L'objectif de ce travail était de comparer l'efficacité de la thoracocentèse répétée versus le drainage thoracique percutané chez les malades adultes souffrant d'empyème thoracique de grande abondance.

**Méthodes:**

Dans cette étude prospective et comparative, 32 patients adultes atteints d'empyèmes thoraciques de grande abondance, répartis en 12 patients dans le groupe thoracocentèse répétée et 20 patients dans le groupe drainage thoracique percutané ont été inclus. Le principal critère de comparaison était la proportion de patients des deux groupes qui étaient décédés dans le service ou transférés en chirurgie (évolution défavorable). Les critères sécondaires de comparaison étaient la durée d'hospitalisation et les complications liées à chacune de ces deux techniques.

**Résultats:**

Les caractéristiques des malades à l'entrée étaient superposables dans les deux groupes. L’évolution défavorable était notée chez 3(25%) malades du groupe thoracocentèse et chez 5(25%) malades du groupe drainage thoracique (P = 1,000). L’échec de la thoracocentèse était noté dans 3 cas et l’échec du drainage thoracique dans 4 cas. Un (5%) patient était décédé dans le groupe drainage et aucun patient n’était décédé dans le groupe thoracocentèse. La durée moyenne d'hospitalisation était de 31,7±22,7 jours chez les patients traités par thoracocentèse versus 29,4±16,7 jours chez les patients traités par drainage thoracique (P = 0,768). Les complications liées à ces techniques étaient retrouvées chez 4(20%) malades traités par drainage et chez 1(8,3%) malade traité par thoracocentèse (P= 0,626).

**Conclusion:**

La thoracocentèse répétée et le drainage thoracique percutané paraissent avoir un taux d’échec et de complications similaire dans le traitement des empyèmes pleuraux de grande abondance.

## Introduction

Les empyèmes thoraciques demeurent malgré le progrès de l'antibiothérapie un sujet d'actualité en pathologie infectieuse thoracique. Ils représentent encore en Afrique subsaharienne 15 à 25% des causes des épanchements pleuraux hospitalisés dans les services de pneumologie [1–3]. Le choix de la technique d’évacuation du pus pleural est toujours un objet de controverse, principalement entre thoracocentèse répétée et drainage thoracique percutané à la phase fibrinoprulente des pleurésies purulentes. Il existe peu d’études comparant directement l'efficacité de ces deux méthodes thérapeutiques [4]. Il nous a paru intéressant de comparer l'efficacité de la thoracocentèse répétée versus le drainage thoracique percutané chez les malades adultes souffrant d'empyème thoracique de grande abondance.

## Méthodes

### Patients

Nous avons conduit une étude prospective, comparative et observationnelle sur une période de 3 ans 2 mois allant d'août 2007 à septembre 2010 parmi les patients adultes âgés de plus de 15 ans hospitalisés dans le service de pneumologie de l'Hôpital Jamot de Yaoundé pour empyème thoracique non tuberculeux à germes communautaires. Le diagnostic d'empyème thoracique a été porté chez les malades présentant des données radio-cliniques d’épanchement pleural liquidien confirmé par une ponction pleurale et en présence d'au moins un des critères suivants:1) liquide pleural franchement purulent à l'examen macroscopique 2) liquide pleural trouble et contenant au moins 70% de polynucléaires neutrophiles altérés. Nous avons seulement inclus les malades présentant des empyèmes thoraciques de grande abondance. L’épanchement pleural était considéré comme de grande abondance quand il occupait au moins la moitié d'un hémithorax.

### Méthodes

**Figure 1:**
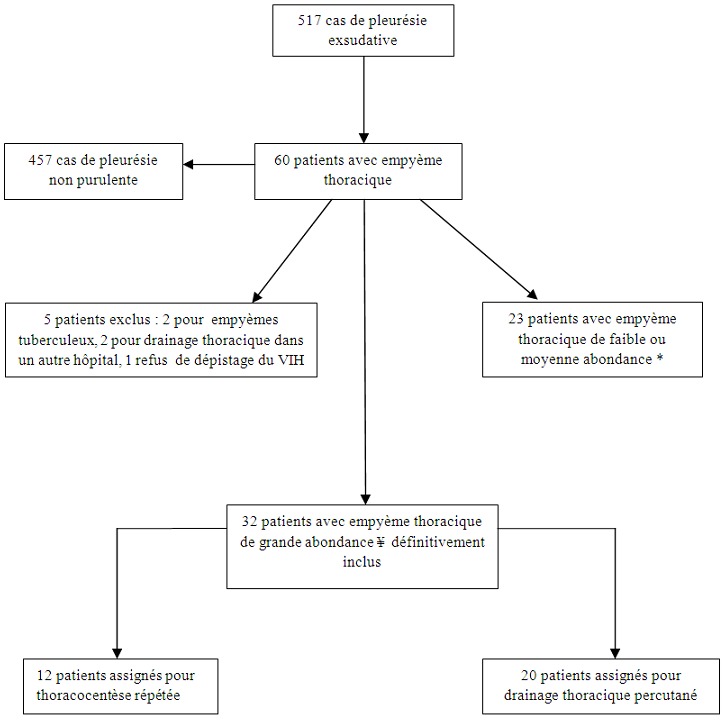
Inclusion des patients dans l’étude

A l'entrée dans le service, tous les malades ont bénéficié d'un examen clinique complet et d'une radiographie standard du thorax avec incidence de face et de profil. Une ponction pleurale a été effectuée sur chaque malade et les caractères macroscopiques du liquide pleural noté. Le taux de protéines, de glucose et de lactico-déshydrogénase(LDH) ainsi que la numération et la formule des éléments leucocytaires de ce liquide ont été déterminés. Le liquide pleural a été également examiné après coloration de Gram pour la recherche des bactéries pyogènes et ensemencé sur milieux aérobie et anaérobie usuels pour culture et identification des germes. La sensibilité des souches bactériennes isolées a été testée par la méthode de dilution. Le liquide pleural était aussi examiné après coloration à l'auramine pour la recherche de BAAR puis ensemencé sur milieu de Lowenstein-Jensen pour la culture et l'identification du bacille de Koch(BK). Tous les patients ont été traités par les antibiotiques non spécifiques associés à une évacuation du liquide pleural et à une kinésithérapie respiratoire. L'antibiothérapie initiale associait chez tous les malades l'amoxicilline/acide clavulanique, un aminoside et le métronidazole. L'antibiothérapie était secondairement adaptée aux données de l'antibiogramme quand la culture du liquide pleural était positive. La durée totale de l'antibiothérapie était de 3 à 6 semaines. Enfin, Les résultats de l'hémogramme, de la sérologie VIH, de la protéine C-réactive ont été extraits du dossier des malades. Pendant la période d'inclusion (Figure 1), 60 patients ont été hospitalisés dans le service pour empyèmes thoraciques. Cinq patients ont été exclus: deux patients présentaient un empyème thoracique tuberculeux, deux patients avaient eu un drainage thoracique dans un autre hôpital et un patient avait refusé le dépistage de l'infection à VIH. Par ailleurs, les 23 patients qui présentaient un empyème occupant moins de la moitié d'un hémithorax n'ont pas été inclus. Deux groupes de patients ont été formés. Le groupe T, composé des patients traités par thoracocentèse répétée et le groupe D composé des patients traités par drainage thoracique percutané. Les patients étaient assignés dans chacun des 2 groupes selon les habitudes du médecin-spécialiste ayant vu le patient à l'entrée dans le service. Ainsi, 32 patients ont été définitivement inclus dans l’étude, répartis en 12 patients dans le groupe T et 20 patients dans le groupe D. Les patients du groupe T avaient une thoracocentèse quotidienne associée à un lavage à la solution salée isotonique. Les ponctions pleurales étaient réalisées à l'aide d'une aiguille de calibre G16. Le lavage pleural consistait à des instillations de 50 à 500 ml de solution salée isotonique qu'on aspirait immédiatement. La procédure était répétée jusqu’à l'obtention d'un liquide pleural clair. Le drainage thoracique percutané chez les patients du groupe D était fait à l'aide d'un seul drain thoracique de calibre 24F placé dans le 4^ème^ ou 5^ème^ espace intercostal de la région latérale de l'hémithorax concerné. Un lavage pleural quotidien semblable à celui utilisé pour la thoracocentèse était également fait à travers le drain thoracique, puis le drain était connecté au bocal de collection du liquide pleural. La fibrinolyse intrapleurale n'avait pas été utilisée chez nos malades. Une radiographie du thorax avec incidence de face et de profil était réalisée après la pose du drain pour vérifier sa bonne position. En outre, tous les patients avaient eu une radiographie du thorax avec incidence de face et de profil après évacuation aussi complète que possible du liquide pleural. Les patients étaient transférés dans le service de chirurgie thoracique pour traitement chirurgical de l'empyème thoracique en cas d’échec de l'une des méthodes thérapeutiques. L’évolution était jugée favorable si tous les critères suivants étaient remplis:1) assèchement de l’épanchement pleural ou réduction du volume de l’épanchement ≥ 5; 70% du volume initial 2) amélioration des signes fonctionnels de la pleurésie purulente et en particulier disparition de la fièvre 3) normalisation de la protéine C-réactive. La pachypleurite séquellaire a été appréciée par la mesure de l'opacité pleurale résiduelle sur la radiographie thoracique de face effectuée 3 mois après la sortie de l'hôpital pour les malades ayant eu une évolution favorable. La distance(en mm) entre la paroi latérale du thorax et le bord interne de l'opacité pleurale au niveau le plus haut de l'hémidiaphragme de l'hémithorax atteint a été mesurée pour la quantification de l'opacité pleurale résiduelle. Si l’épanchement pleural était enkysté latéralement, la quantification de l'opacité résiduelle a été faite par la mesure de la plus grande distance horizontale de cette opacité. Une opacité pleurale résiduelle a été considérée comme étant significative si elle mesurait au moins 10 mm [5]. Le principal critère de comparaison était l’évolution défavorable au cours du traitement. L’évolution défavorable incluait le transfert en chirurgie du fait de l’échec de l'une des méthodes thérapeutiques et le décès au cours de l'hospitalisation dans notre service. Les critères secondaires de comparaison étaient les complications liées aux 2 techniques thérapeutiques et la durée d'hospitalisation. Ces complications pouvaient être: la surinfection pleurale nosocomiale, la fistule bronchopleurale ou pleuro-cutanée et le pneumothorax iatrogène. L’étude a eu une clairance éthique du Comité National d'Ethique du Cameroun et une autorisation administrative de recherche des autorités de l'Hôpital Jamot de Yaoundé. De même, tous les malades inclus ont donné leur consentement éclairé avant l'inclusion dans l’étude.

Les proportions ont été comparées par la méthode de X^2^avec calcul de la probabilité exacte de Fischer quand il s'avérait nécessaire et les variables quantitatives par le test non paramétrique de Mann-Whitney. Une différence a été considérée comme significative si P < 0,05.

## Résultats

### Population d’étude

Trente deux patients adultes atteints d'empyèmes thoraciques de grande abondance ont été inclus dans notre étude. Des 32 patients inclus, 12 patients ont été assignés dans le groupe T et 20 patients dans le groupe D. Les caractéristiques démographiques et cliniques (Table 1) ainsi que les caractéristiques radiographiques et hématologiques (Table 2) des patients à l'entrée dans le service étaient similaires dans les deux groupes.

**Table 1:** Principales caractéristiques démographiques et cliniques initiales des patients des deux groupes des patients

Caractéristiques démographiques ou cliniques	Groupe T (n = 12)	Groupe D (n = 20)	P
**Sexe-n(%)**			
Homme	7(58,3)	14(70)	0,702
Femme	5(41,7)	6(30)	0,702
Age, ans (M, ET)	40,3±19,3	40,9±14,0	0,744
**Facteurs favorisants-n(%)**			
Infection à VIH	6(50)	8(40)	0,580
Diabète sucré	1(8,3)	2(10)	1,000
Autres	2(16,7)	4(20)	1,000
Aucun	3(25)	6(30)	0,625
**Symptômes-n(%)**			
Toux	11(91,7)	20(100)	0,375
Douleur thoracique	11(91,7)	16(80)	0,626
Dyspnée	10(83,3)	20(100)	0,133
Fièvre	12(100)	12(60)	0,010
Amaigrissement	10(83,3)	16(80)	1,000
Délai d'installation des symptômes (M, ET)	13,6±19,9	38,4±39,7	0,326
Antibiothérapie avant prise en charge-n(%)	7(58,3)	13(65)	0,724

Groupe T: patients traités par thoracocentèse répétée, Groupe D: patients traités par drainage thoracique percutané, M: moyenne, ET: écart-type

**Table 2:** Caractéristiques radiographiques et hématologiques initiales des patients des deux groupes

Caractéristiques radiographiques et hématologiques	Groupe T (n = 12)	Groupe D (n = 20)	P
**Radiographie du thorax-n(%)**			
Epanchement pleural droit	7(58,3)	11(55)	0,854
Epanchement enkysté	4(33,3)	3(15)	0,218
Pneumothorax associé	2(66,7)	6(30)	0,675
Condensation pulmonaire	10(83,3)	14(70)	0,675
Abcès du poumon	1(8,3)	1(5)	1,000
**Hématologie**			
Leucocytes, mm3 (M, ET)	15905±11359,2	13660±6502,6	0,803
Taux d'hémoglobine, g/l (M, ET)	10,4±3,1	9,8±2,7	0,774

Groupe T: patients traités par thoracocentèse répétée, Groupe D: patients traités par drainage thoracique percutané, M: moyenne, ET: écart-type

Le Table 3 présente les résultats de l'analyse bactériologique du pus pleural des patients du groupe T et du groupe D. La culture du liquide pleural à la recherche des germes pyogènes étaient positive chez 6(50%) patients du groupe T et chez 11 (55%) patients du groupe D (P = 0,784). Streptococcus pneumoniae était le germe le plus fréquemment isolé dans les deux groupes avec 3(42,9%) cas dans le groupe T et 5(41,7%) cas dans le groupe D.

**Table 3:** Bactériologie du liquide pleural des deux groupes des patients

Bactériologie	Groupe T (n = 12)	Groupe D (n = 20)
**Culture positive-n(%)**	6(50)	11(55)
**Germes isolés-n**		
*Streptococcus pneumoniae*	3	5
*Streptococcus pyogènes*	2	1
*Streptococcus sp*	0	2
*Staphylococcus aureus*	1	0
*Haemophilus influenzae*	0	2
*Citrobacter freundi*	0	1
*Peptococcus sp*	1	1

Groupe T: patients traités par thoracocentèse répétée, Groupe D: patients traités par drainage thoracique percutané

### Principal critère de comparaison

Le Table 4 compare le devenir des malades des deux groupes au cours de l'hospitalisation dans notre service. L’évolution défavorable était notée chez 3(25%) des 12 malades du groupe T et chez 5(20%) des 20 malades du groupe D (P= 1,000). L’échec de la thoracocentèse était noté dans 3 cas et l’échec du drainage thoracique dans 4 cas. Un (5%) patient était décédé dans le groupe D et aucun décès n'a été enregistré dans le groupe T.

**Table 4:** Devenir des patients des deux groupes

Devenir	Groupe T (n = 12)	Groupe D (n = 20)	P
Durée d'hospitalisation, j (M, ET)	31,7±22,7	29,4±16,7	0,922
Nombre de ponctions pleurales réalisées (M,ET)	6,5±5,1	-	
Durée du drainage thoracique, j (M, ET)	-	10,1±3,4	
Complications au cours de l'hospitalisation-n(%)	1(8,3)	4(20)	0,626
Pneumothorax iatrogène	1(8,3)	2(10)	
Surinfection pleurale nosocomiale	0(0)	2(10)	
Evolution défavorable	3(25)	5(25)	1,000
Echec	3(25)	4(20)	
Décès	0(0)	1(5)	
Pachypleurite résiduelle*-n(%)	1/9(11,1)	1/15(6,7)	1.000

Groupe T: patients traités par thoracocentèse répétée, Groupe D: patients traités par drainage thoracique percutané, j = jours, M = moyenne, ET = écart-type

* Evaluer 12 semaines après la sortie de l'hôpital

### Critères secondaires de comparaison

La durée d'hospitalisation était similaire dans les deux groupes de nos patients avec une durée d'hospitalisation de 31,7±22,7 jours dans le groupe T et de 29,4±16,7 jours dans le groupe D (P = 0,626). Dans notre étude, la fréquence des complications intra-hospitalières était de 20% dans le groupe D contre 8,3% dans le groupe T avec une différence non significative. Ces complications comprenaient 1(8,3%) cas de pneumothorax dans le groupe T, de 2(10%) cas de pneumothorax dans le groupe D et de 2(10%) cas de surinfection pleurale à Pseudomonas aeruginosa dans le groupe D. La pachypleurite significative a été retrouvée chez 1 malade dans chaque groupe (P = 1.000) (Table 4).

## Discussion

Il existe actuellement et à notre connaissance peu d’études comparant directement l'efficacité de la thoracocentèse répétée à celle du drainage thoracique percutané dans les empyèmes thoraciques. Ces deux méthodes sont utilisées par plusieurs équipes à la phase fibrino-purulente de l'infection pleurale. La taille relativement faible de notre échantillon est en partie liée au fait que seuls les patients ayant un épanchement pleural purulent de grande abondance ont été inclus. Cela nous a permis d'avoir une homogénéité entre les deux groupes de patients.

Les signes radio-cliniques retrouvés chez nos patients atteints d'empyèmes thoraciques traités par thoracocentèse répétée ou par drainage thoracique percutané sont ceux habituellement décrits pour cette affection. Le taux de positivité de la culture du liquide pleural était de 50% chez nos patients traités par thoracocentèse répétée et de 55% chez ceux qui étaient traités par drainage thoracique percutané. Ce taux est inférieur au taux variant de 60 à 85% rapporté par la plupart des auteurs [6–9]. Ce faible taux de positivité de la culture du liquide pleural chez nos malades peu être expliqué par la prise fréquente d'antibiotiques par nos malades avant leur admission dans le service. En effet, 62,5% de nos malades avaient pris les antibiotiques avant leur admission dans le service.

Cette étude a montré que la thoracocentèse répétée et le drainage thoracique percutané ont une efficacité équivalente dans le traitement de l'empyème thoracique. Le taux de succès de ces deux méthodes thérapeutiques dans notre série était de 75%. Storn et al [4]. avaient trouvé en 1992 un taux de succès de 91% pour la thoracocentèse et un taux de 21% pour le drainage thoracique. En effet, l’étude de ces derniers auteurs était rétrospective et les malades initialement traités par drainage thoracique étaient hospitalisés dans un service de chirurgie et ceux traités par thoracocentèse étaient hospitalisés dans un service de médecine. Ce qui rend la comparaison des deux groupes des malades de l’étude de Storn et al. difficilement interprétable. Dans la plupart des études au cours desquelles le drainage thoracique est la méthode utilisée, le taux de succès varie de 69% à 86% [6, 8, 10–12]. Aucun malade traité par thoracocentèse n’était décédée et le taux de mortalité chez les malades traités par drainage thoracique était de 5%. Le taux de mortalité par empyème pleural varie selon les données récentes de la littérature de 4% à 29% [1, 8, 10, 11, 13]. Ce faible taux de mortalité trouvée dans notre étude peut s'expliquer par le jeune âge de nos malades et par la faible fréquence des tares classiquement responsables de la mortalité (diabète sucré, affection cardiovasculaire, cancer) chez les malades souffrant d'empyèmes pleuraux.

L'analyse des critères secondaires de comparaison dans cette étude montre que la durée d'hospitalisation était similaire chez les malades traités par thoracocentèse et chez ceux traités par drainage thoracique. Les complications liées aux deux méthodes thérapeutiques utilisées chez nos malades étaient plus fréquentes chez les malades traités par drainage thoracique percutané que chez les malades traités par thoracocentèse répétée, mais cette différence n’était pas significative. Particulièrement, la surinfection pleurale nosocomiale n'a été retrouvée que chez nos malades traités par drainage thoracique et avait concerné 10% de ces 20 malades. Storn et al [4]. avaient trouvé une surinfection pleurale nosocomiale significativement plus fréquente chez les patients traités par drainage thoracique. Le drain thoracique semble augmenter la fréquence des surinfections pleurales nosocomiales chez les malades ayant un empyème thoracique. Le coût moyen de chacune des deux méthodes thérapeutiques n'a pas été évalué dans notre étude. Cette évaluation nous aurait permis au cas o[ugrave] l'une des méthodes s’était révélée moins coûteuse de pouvoir la proposer comme méthode de choix dans un pays aux ressources limitées comme le Cameroun.

## Conclusion

En conclusion, dans cette étude, la thoracocentèse répétée et le drainage thoracique percutané paraissent avoir un taux d’échec similaire dans le traitement des empyèmes pleuraux de grande abondance à Yaoundé. La durée d'hospitalisation n'est réduite par aucune des deux méthodes thérapeutiques mais la surinfection pleurale nosocomiale n'a été retrouvée que chez les patients traités par drainage thoracique percutané. Des études prospectives randomisées à plus grande échelle doivent être réalisées afin de confirmer ces données.

## References

[1] Kuaban C, Gonsu-Fosting J, Nlend R, Mbakop A, Amana JP (1993). Les épanchements pleuraux à Yaoundé(Cameroun) Etude étiologique de quatre-vingt-quatre cas. Sem Hôp Paris..

[2] Dangra AY, Gbadoe AH, Edorh TK, Prince-David P, Tidjani O, Sadzo DH (2004). Fréquence et impact de l'infection au virus de l'immunodéficience humaine chez les patients souffrant de pleurésies bactériennes à Lomé. Med Mal Infect..

[3] Diallo S, Hassan M, Sissoko F, M'baye O, Gomez P (2006). Etiologies des pleurésies dans le service de pneumologie du point G. Mali Med..

[4] Storm HK, Krasnik M, Bang K, Frimodt-Moller N (1992). Treatment of pleural empyema secondary to pneumonia: thoracocentesis regimen versus tube drainage. Thorax..

[5] Han DH, Song JW, Chung HS, Lee JH (2005). Resolution of residual pleural disease according to time course in tuberculous pleurisy during and after the termination of antituberculosis medication. Chest..

[6] Alfageme I, Muñoz F, Peña N, Umbría S (1993). Empyema of the thorax in adults Etiology, microbiologic findings, and management. Chest..

[7] Brook I, Frazier EH (1993). Aerobic and anaerobic microbiology of empyema: a retrospective review in two military hospitals. Chest..

[8] Maskell NA, Davies CW, Nunn AJ, Hedley EL, Gleeson FV, Miller R (2005). First Multicenter Intrapleural Sepsis Trial (MIST1) Group UK Controlled trial of intrapleural streptokinase for pleural inf. N Engl J Med..

[9] Lin YC, Chen HJ, Liu YH, Shih CM, Hsu WH, Tu CY (2008). A 30-month experience of thoracic empyema in a tertiary hospital: emphasis on differing bacteriology and outcome between the medical intensive care unit (MICU) and medical ward. South Med J..

[10] Chen KY, Hsueh PR, Liaw YS, Yang PC, Luh KT (2000). A 10-year experience with bacteriology of acute thoracic empyema: emphasis on Klebsiella pneumoniae in patients with diabetes mellitus. Chest..

[11] Simmers TA, Jie C, Sie B (1999). Minimally invasive treatment of thoracic empyema. Thorac Cardiovasc Surg..

[12] Davies CW, Kearney SE, Gleeson FV, Davies RJ (1999). Predictors of outcome and long-term survival in patients with pleural infection. Am J Respir Crit Care Med..

[13] Ahmed RA, Marrie TJ, Huang JQ (2006). Thoracic empyema in patients with community-acquired pneumonia. Am J Med..

